# Eco-friendly alternatives to seed chemical coating: agro-industrial byproducts as seed treatments with long-term effects on growth and reproduction

**DOI:** 10.1186/s40643-025-01000-5

**Published:** 2026-01-24

**Authors:** Joy Jacklin Jayaseelan, Emilly Draru, Govindegowda Priyanka, Keerthana Yeduguru Reddy, Nurit Novoplansky, Ilan Chertok, Elena Poverenov, Gideon Grafi

**Affiliations:** 1 French Associates Institute for Agriculture and Biotechnology of Drylands, Jacob Blaustein Institutes for Desert Research, Ben-Gurion University of the Negev, 84990, Midreshet Ben Gurion, Israel; 2 Department of Food Science, Agro-Nanotechnology and Advanced Materials Research Center, Agricultural Research Organization, The Volcani Institute, Rishon Lezion, Israel; 3 The Robert H. Smith Faculty of Agriculture, Food and Environment, Biochemistry, and Food Sciences, The Hebrew University of Jerusalem, 76100 Rehovot, Israel

**Keywords:** Agro-industrial byproducts and wastes, Wheat bran, Wine pomace, Brewer’s spent grain, Garlic peels, Seed coating, Circular economy, Wheat growth and reproduction, Sustainable agriculture.

## Abstract

Synthetic chemical seed treatments, while effective, often raise significant environmental and health concerns. These concerns stem from the use of hazardous chemicals such as fungicides and insecticides that, besides posing risks to workers, have broader environmental impacts. These hazardous chemicals can leach into the soil and water systems, disrupting ecosystems, harming beneficial organisms, and entering the food chain. Agro-industrial byproducts/wastes (AIBWs) represent an abundant, environmentally friendly resource with potential for seed treatments. We focused on AIBWs that are produced in enormous amounts and do not pose potential hazards since they are commonly used to feed animals as well as food additives for humans, including wheat bran (WB), wine pomace (WP), and brewer’s spent grain (BSG). We investigated the effects of imbibing wheat seeds in water-soluble extracts of AIBWs or coating seeds with a biopolymer supplemented with AIBW substances on wheat growth and reproduction. As controls, we used water-soaked (WS) and non-soaked (NS) seeds, as well as chemically Celest Top-coated seeds. Petri dish assays showed that seeds imbibed in AIBW extracts exhibited enhanced post-germination growth as compared to NS seeds. Thus, while 81% of NS seedlings produced up to 3 seminal roots (SRs), 84% of WB and 64% of Celest Top seedlings produced 4 and 5 SRs. Net-house experiments revealed that Celest Top and AIBW extracts had a positive effect on reproduction as compared to NS, displaying 17.4%, 14.5%, 30.3%, and 34.3% increases in grain weight per spike in Celest Top, WB, GP, and WP, respectively. Metabolic analysis of seeds derived from treated plants revealed variation in metabolite profiles with a notable increase in the amino acid tryptophan. We utilized the nature-sourced polysaccharide carboxymethylcellulose (CMC) to coat seeds with AIBW substances derived from GP, referred to as CMC-GP. The results indicated that CMC-GP and Celest Top enhanced root growth, displaying 2- and 1.5-fold increases in fresh and dry weight, respectively, as compared to NS and CMC-coated seeds. Thus, AIBWs appear to provide cost-effective, eco-friendly alternatives to the hazardous chemical seed coatings, whether applied *via* imbibition or coating, while aiding in waste valorization within the circular economy.

**Graphical abstract:**

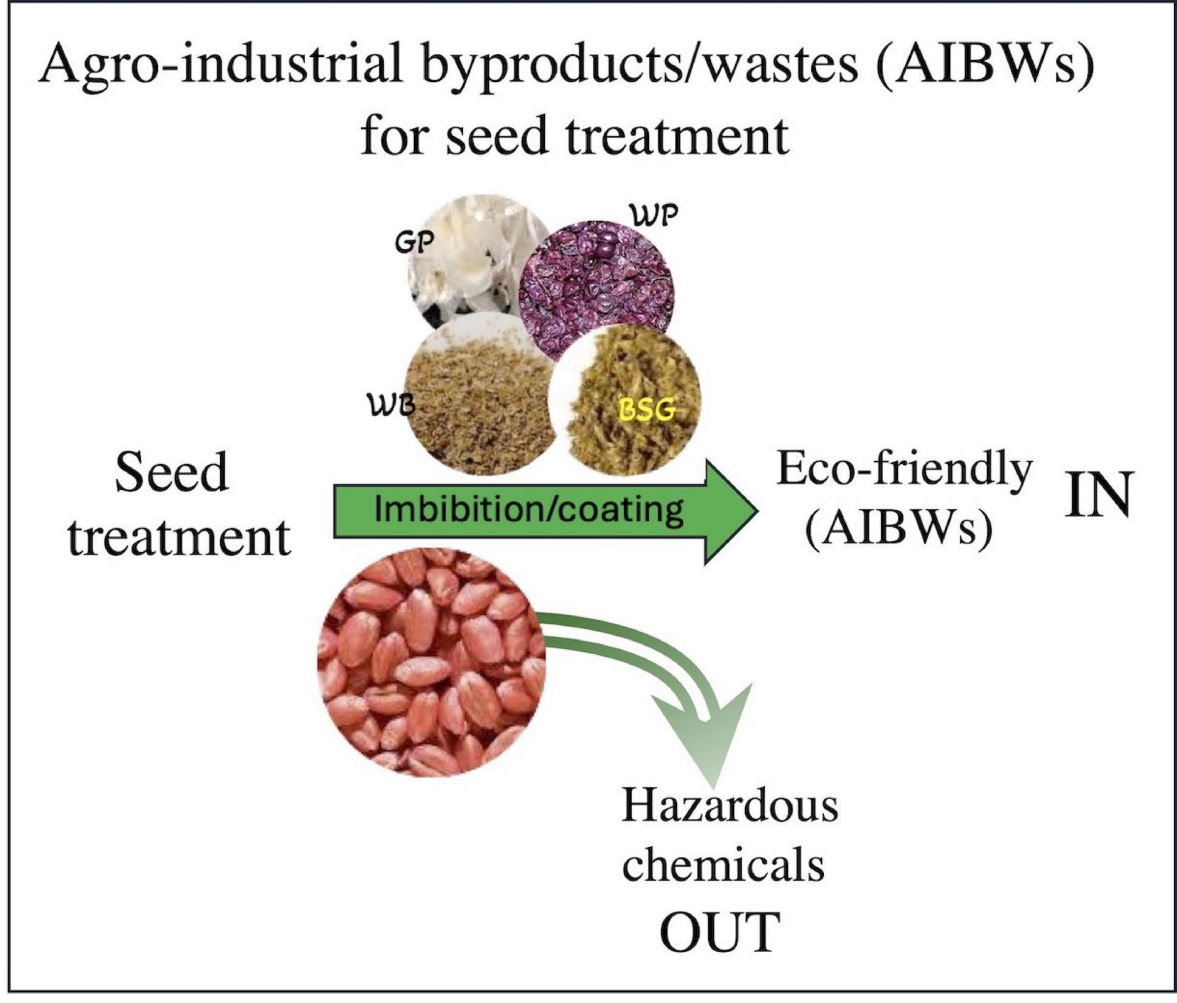

**Supplementary Information:**

The online version contains supplementary material available at 10.1186/s40643-025-01000-5.

## Introduction

The rapidly growing global population presents a considerable challenge to achieving food security on a global scale. Addressing this increasing demand requires a comprehensive strategy that includes the sustainable enhancement of agricultural practices, reducing food loss and waste, and improving the resilience of cropping systems. A fundamental aspect of successful agriculture is the quality of the seeds utilized. High-quality seeds, characterized by superior germination rates, vigorous seedling establishment, and enhanced tolerance to biotic and abiotic stresses, are fundamental for maximizing crop yields and ensuring a stable food supply (Wimalasekera 2015; Ren et al. 2023). Synthetic chemical seed treatments, while often effective, raise significant environmental and health concerns (Curl et al. 2020). These concerns include potential risks to agricultural workers who handle treated seeds (Curl et al. 2020), as well as broader environmental impacts. These chemicals can leach into the soil and water systems, disrupting ecosystems, harming beneficial organisms, and potentially entering the food chain (Schmuck and Lewis 2016; Margaoan et al. 2025). Therefore, the development of sustainable and environmentally friendly alternatives to conventional seed treatments is a critical requirement (Ranganathan and Groot 2023; Groff et al. 2025).

One promising avenue for achieving this goal lies in the utilization of agro-industrial byproducts and wastes (AIBWs). The generation of AIBWs is an inherent and substantial consequence of modern agricultural practices and food processing industries (Sadh et al. 2018). The immense quantity of these byproducts, ranging from crop residues like stalks and husks to processing wastes such as peels, pomace, and spent grains, presents a significant, complex, and diverse challenge. If mismanaged, these AIBWs can become a major source of environmental pollution, contributing to issues such as soil and water contamination, air pollution from burning or decomposition, and increased landfill burden (Sadh et al. 2018; Barbera 2020; Kumar et al. 2023).

AIBWs are complex matrices rich in a wide variety of valuable compounds (Sadh et al. 2018; Priyanka et al. 2024). A detailed and comprehensive analysis of the composition of different AIBWs is crucial to enable efficient sorting and targeted utilization of these resources for various agro-industrial applications (Priyanka et al. 2024). By effectively utilizing AIBWs (e.g., wine pomace and brewery’s spent grain), we can mitigate the environmental problems associated with their disposal, simultaneously generating new economic opportunities and promoting a more sustainable and resource-efficient agricultural system (Apprich et al. 2014; Bianco et al. 2024; Buchmann et al. 2025; Sisti et al. 2021).

The present study focuses on exploring the potential of selected AIBWs as sustainable supplemental materials for wheat (*Triticum aestivum L*.) seeds. Wheat is a staple food crop globally, providing a significant portion of the world’s calorie and protein intake. Improving wheat production is therefore crucial for ensuring global food security. The chosen AIBWs—wheat bran (WB), garlic peels and stalks (GP), brewer’s spent grain (BSG), and wine pomace (WP)—are considered safe and environmentally friendly resources. They are generated in large quantities and utilized for animal feed as well as food additives for human consumption; therefore, no potential risks are anticipated in their application. The WB represents approximately 25–30% of the wheat grains, and its yearly production is about 150–200 million tons globally. It is rich in bioactive compounds, dietary fibers, and proteins and commonly used to feed animals and in the human food industry (Onipe et al. 2015; Prückler et al. 2014; Katileviciute et al. 2019; Priyanka et al. 2024). Commercial breweries produce about 40 million metric tonnes of BSG annually, which is used for human consumption after processing, as it is a nutrient-rich source of fiber and proteins (Aradwad et al. 2025; Nyhan et al. 2023; Chattaraj et al. 2024). The global wine industry produces approximately 10.5 to 14 million tons of WP annually. This byproduct is rich in bioactive compounds, including a variety of polyphenols, dietary fibers, and organic acids, which can be utilized in multiple industries such as food, cosmetics, pharmaceuticals, and bioenergy (Karastergiou et al. 2024; van Wyk et al. 2025). The global production of GP is approximately 3.7 million tons annually (Sunanta et al. 2023). Most garlic waste is disposed into landfills or burned, which negatively affects the environment (Kallel and Ellouz Chaabouni 2017). Garlic waste can be used as a source for antimicrobial substances, biostimulants, and the production of cellulose derivatives, which can be used as nanofillers for polymer matrices, as well as for soil amendment and bioenergy (Kallel and Ellouz Chaabouni 2017; Naqvi et al. 2020; Singiri et al. 2022; Moreno et al. 2020; Priyanka et al. 2024).

The major goal of the study is to assess the biological significance of AIBWs as potential source for beneficial substances for wheat seed treatment via seed imbibition to enhance plant growth and development. In addition, we utilized the nature-sourced polysaccharide carboxymethylcellulose (CMC) to coat seeds with AIBW substances. As controls, we used water-soaked and non-soaked seeds, as well as CMC- and Celest Top-coated seeds. Our study provides valuable data highlighting the beneficial and enduring impacts of seed treatment by AIBWs on wheat plant performance from germination to vegetative growth and reproduction.

## Materials and methods

### Preparation of AIBW extracts and seed imbibition and coating

Extracts were prepared from four grounded AIBWs, namely, WB, GP, BSG and WP. Fifty grams (50 g) of each byproduct were individually extracted in 1.5 L except for GP which extracted with 2 L of double-distilled water, due to its mucilaginous nature. Extraction was carried out at 4 °C with constant stirring for 1 h, homogenates were filtered through 300 nm nylon mesh followed by centrifugation (8000 × g for 30 min at 18 °C). The clear supernatant was collected (this is a 100% extract) and store at -20 °C until used. The 50% and 25% extracts were prepared by diluting the 100% extract with double-distilled water. Commercially available wheat seeds (cultivar ‘Gadish’) were imbibed in AIBW extracts as follows: Five hundred grams (500 g) of wheat seeds were soaked and allowed to imbibe in varying concentrations (100%, 50%, and 25%) of the prepared WB, GP, BSG, and WP extracts or in double distilled water (WS), which was used as a control. The seeds were soaked for 12 h at 4 °C with continuous stirring to facilitate optimal imbibition. After the imbibition period, the seeds were drained, placed on blotting paper to absorb excess moisture, and subsequently completely air-dried in a sterile chamber. We used Celest Top-coated seeds as a reference control for imbibed seeds.

Seed coating was performed as follows: Powder of the lyophilized GP extract was dissolved in 700 mL of 1% (w/w) carboxymethylcellulose (CMC) solution. The final suspensions consisted of 1% CMC containing 10 mg/mL of GP substances (CMC-GP). Wheat seeds (500 g) were soaked in CMC–GP solution for 10 min. The seeds were mechanically mixed with a spatula to ensure uniform coating. The coated seeds were then passed through a strainer to remove excess solution and placed on a clean paper towel-lined tray. The trays were incubated at 37 °C overnight to allow coated seeds to dry. Coated seeds were packaged and stored at -18 °C until used.

### Seed storage and germination and postgermination analysis

To assess the long-term viability and germination potential of the imbibed seeds, a storage experiment was conducted. Post-drying, the imbibed seeds from each treatment were divided into three equal portions. These portions were then stored under different temperature conditions: room temperature (approximately 24 °C), 4 °C, and − 20 °C. The effect of these varying storage temperatures on seed viability was evaluated immediately after drying or after 1- and 6-months storage. Germination and post-germination tests were performed in Petri dishes lined with moist blotting paper (20 seeds in a plate). Four replicates were used per treatment, totaling 80 seeds per treatment. WS, NS and Celest Top-coated seeds were used as controls. Monitoring and Data Collection: Petri dishes were placed in a growth room under controlled conditions (photoperiod light: dark 12 h:12 h; temperature 24+/-2). Germination was monitored every 24 h for a total period of 72 h. Following germination, the fresh weight of the seedings, root length, shoot length, and the number of seminal roots were recorded.

### Net house experiments

A net house experiment was conducted to assess the full growth cycle and yield parameters under semi-controlled conditions, allowing for harvest. This experiment utilized 10-liter capacity pots filled with field-type soil (Alluvial soil). Nine treatments were prepared, including seeds imbibed in 50% concentration of each AIBW, WS, NS and Celest Top-coated seeds. Three pots were allocated per treatment, serving as replicates. Twenty seeds were sown in each pot. Irrigation was provided daily for 10 min via a drip system (2 L/hour dripers). Fertilization with NPK (4% nitrogen, 2.5% phosphorus, and 6% potassium, 0.5% Mg, 2% Ca) was administered once a week. The experiment began on December 18th and concluded on May 4th, allowing the plants to reach full maturity and harvest (Data on Max/Min temperature during the growing period is given in Fig. 4A). Data was collected at early stage of development (mid-experiment, January 25th, 2025) one pot from each treatment was destructively sampled for the analysis of growth parameters, including seminal root production, leaf length, shoot length, and fresh and dry weights. The remaining two pots per treatment were maintained until harvest, at which point seed weight, number of seeds per spike, spike length, and final shoot length were measured.

### Metabolic analysis

Metabolic analysis was performed on wheat seeds harvested from net house experiments from plants derived from NS, WS and Celest Top-coated seeds or AIBW extract-treated seeds of WB, GP, WP, BSG or mixed extracts of AIBW (Im4 × 25). Gas chromatography–mass spectrometry (GC–MS) was used to quantify primary metabolites in four biological replicates, essentially as described (Lisec et al. 2006). Briefly, wheat seeds were ground to a fine powder using a bead beater, and the powdered material was used for metabolomic extraction as described previousely (Priyanka et al. 2024). PCA, ANOVA, Student’s t-tests, and hierarchical clustering were performed using MetaboAnalyst 6.0 (Xia and Wishart 2016).

## Results

### Effect of imbibition of wheat seeds in AIBW extracts on their germination and post-germination growth: Petri dish assays

**Fig. 1:**
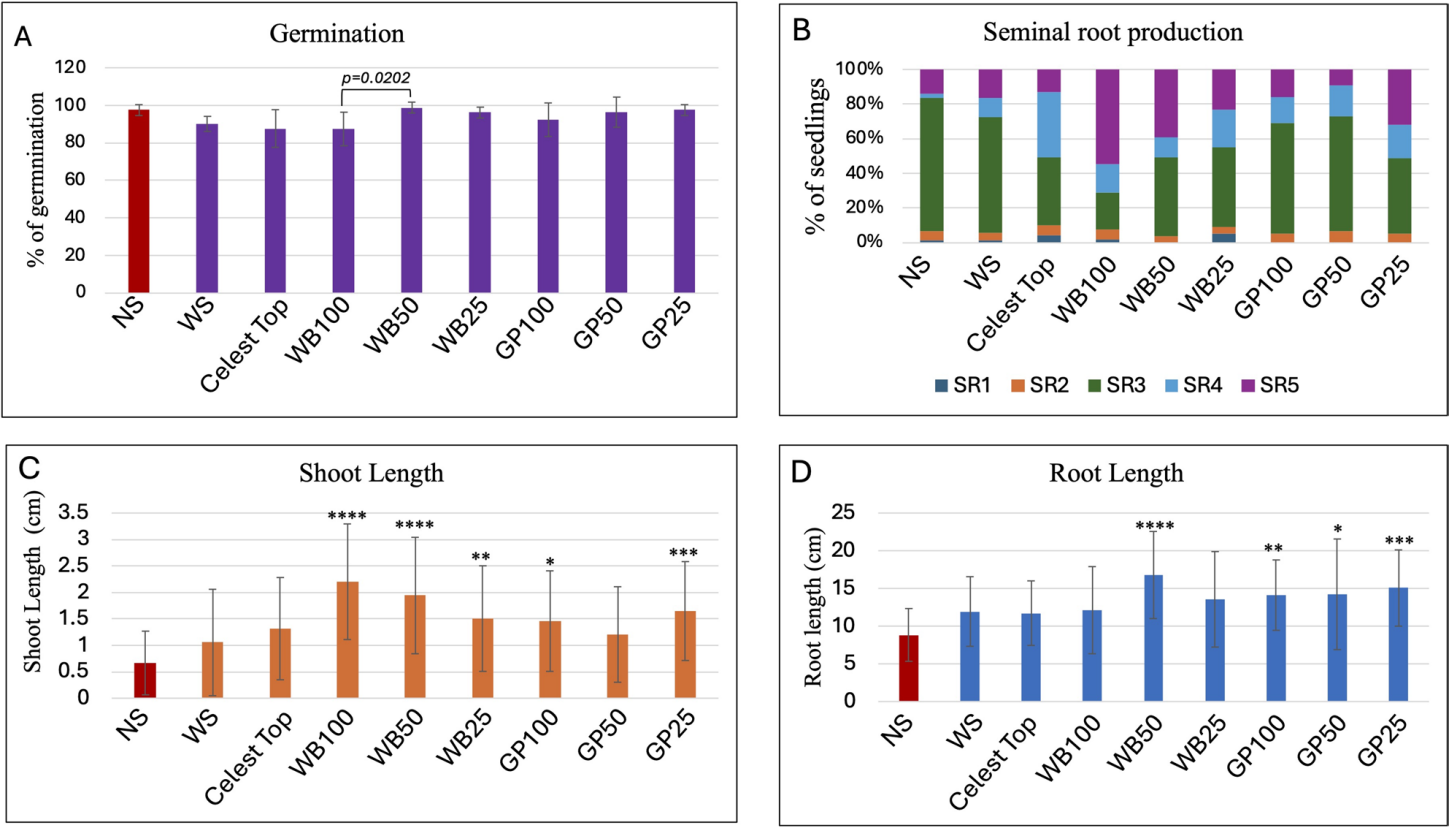
The effect of seed imbibition in AIBW extracts on wheat seed germination and post-germination growth: Petri dish experiment. **A** Germination percentages of treated and control seeds after 72 h. Note, significant difference in germination was found between WB100 and WB50. Effects of AIBW on wheat seedling growth. **B** Effect of AIBWs on seminal root (SR) growth. The proportion of wheat seedlings developing various numbers of seminal roots (SR1 to SR5) is presented in a stacked column. **C** Effect of AIBWs on shoot length as compared to Control NS (red column). **D** Effect of AIBWs on root length as compared to Control NS (red column). Statistically significant differences between control NS and the examined AIBWs are indicated by asterisks. The *p* value was determined using Student’s unpaired t-test (GraphPad software). *p < 0.05; **p < 0.01; ***p < 0.001; ****p < 0.0001

Wheat seeds were imbibed in extracts of WB (wheat bran) and GP (garlic peels) at different concentrations and compared with NS (non-soaked), WS (water-soaked), and Celest Top-coated seeds. Seeds were placed in Petri dishes on wet filter papers and recorded after 72 h. The germination data shown in Fig. 1A reveals that wheat seed germination essentially was not affected significantly following imbibition in AIBW extracts, and treated seeds showed germination ranging from 87.5% in Celest Top and WB100 to 97.5% in control NS and WB50 (Fig. 1A). Statistical analysis revealed a significant difference in germination percentage between WB100 and WB50 (*p* = 0.0202). All other comparisons were not statistically significant.

The effect of AIBWs on the growth parameters of wheat seedlings was monitored after 72 h. The number of seminal roots and their proportion among all seedlings was calculated for each treatment (Fig. 1B). The results indicate that across all treatments, the most common number of seminal roots is three (SR3), followed by SR4 and SR5. Both NS and WS treatments exhibit similar distributions, with SR3 being the most common and moderate proportions of SR5. For the Celest Top treatment, there is a noticeable shift towards SR4, with a decrease in SR3. WB treatments, particularly at higher concentrations (WB100), significantly increased the proportion of higher-order seminal roots (SR4 and SR5). GP treatments show milder effects, with a modest increase in SR5 observed in the GP25 treatment, suggesting that WB, especially at higher concentrations, promotes the development of more seminal roots, which could enhance nutrient and water uptake, thereby potentially enhancing plant performance across diverse conditions.

The effect of WB and GP on shoot length is shown in Fig. 1C. The statistical analysis from the unpaired T-test of wheat shoot lengths following various treatments revealed significant differences in growth outcomes. Seeds soaked in WB at concentrations of 100%, 50%, and 25% all showed significant increases in shoot length compared to the control WS, with the 100% concentration being the most effective. Similarly, GP at 100% and 25% concentrations significantly enhanced shoot length, while the 50% concentration did not yield a statistically significant difference from the control. In contrast, the Celest Top and the control NS were the least effective, with the latter showing a significant decrease in shoot length. These results suggest that soaking seeds in WB and GP extracts can significantly promote wheat shoot growth in a manner dependent on concentration and the type of extract.

Analysis of root length showed (Fig. 1D) variable effects of AIBW extracts as compared to water-soaked seeds. Accordingly, imbibition of seeds in water confers an apparent advantage over non-soaked seeds (Control NS). Conversely, the WB50, GP100, GP50, and GP25 all showed significantly higher root lengths compared to the control WS, indicating that these treatments enhanced root growth. No significant differences in root length were observed between the control WS and the Celest Top or WB100 treatments.

### Determination of the optimal conditions for storage (temperature, period) of dried imbibed seeds

We sought to determine the most suitable storage condition for imbibed seeds that allows maintaining their viability and proper growth. Since the effectiveness of seed treatments depends not only on the bioactive compounds applied but also on their stability during storage, it was essential to assess how different storage environments influenced seed performance. To address this, seeds imbibed in various AIBW extracts were dried and stored under three temperature regimes: room temperature (RT), 4 °C, and − 20 °C for one month and six months. Following storage, the seeds were evaluated for germination percentage and post-germination traits, including seminal root number, total root length, and shoot length. This approach allowed for a direct comparison of both storage duration and temperature, thereby providing insights into which conditions best preserve the beneficial effects of AIBW seed treatments.

### The effect of one month storage on germination and post germination growth of AIBW-imbibed seeds

**Fig. 2:**
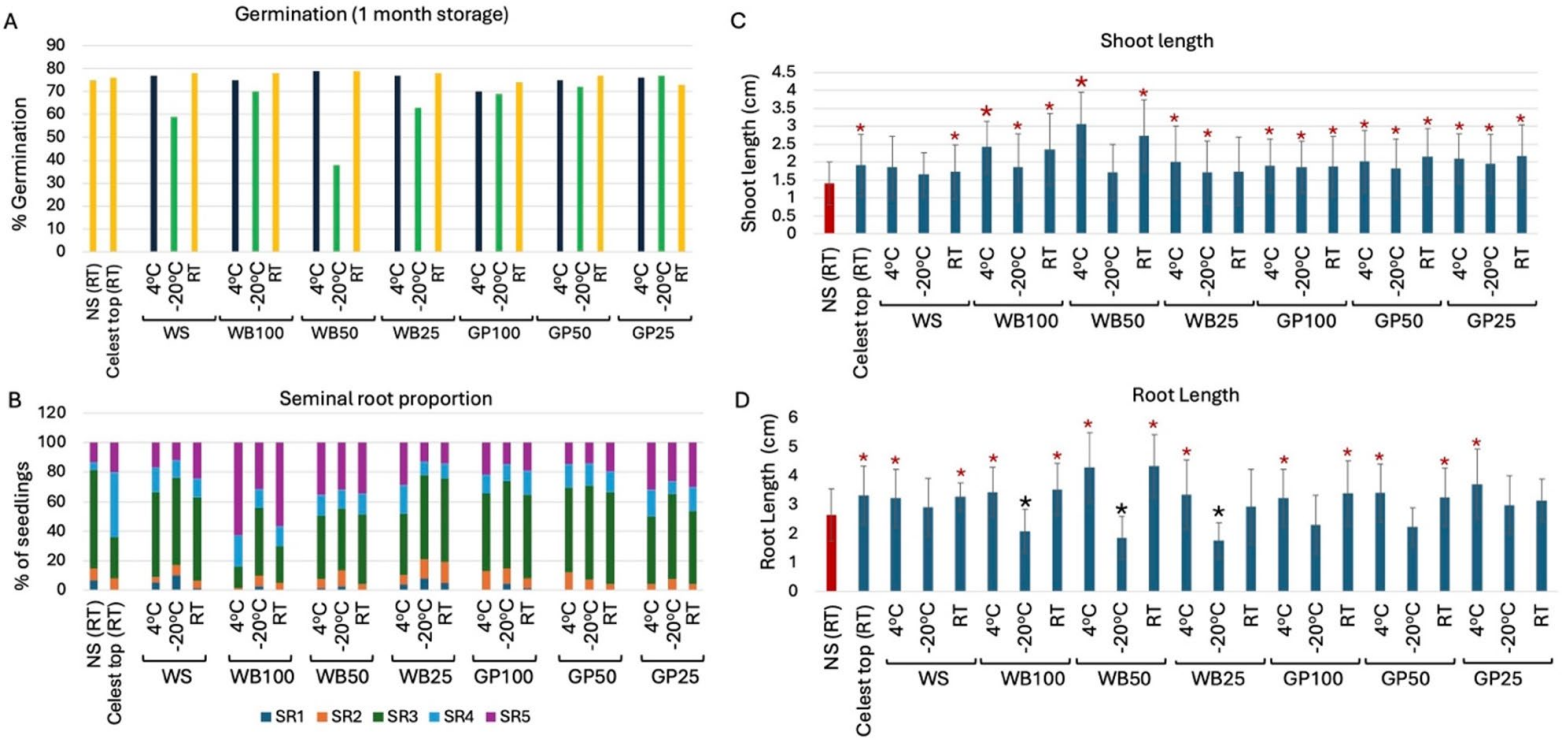
Effects of AIBWs (WB and GP) on germination and seminal root (SR) production after 1 month storage at RT, 4 °C and − 20 °C. Non-soaked (NS) and Celest Top-coated seeds were used as controls. **A** Germination percentages recorded after 72 h. **B** Effect of AIBWs on (SR) production. The proportion of wheat seedlings developing various numbers of seminal roots (SR1 to SR5) is presented in a stacked column. **C**, **D** Effects of various concentrations of AIBWs (WB and GP) on shoot and root length of 72 h seedlings derived from imbibed, dried seeds after 1 month storage at RT, 4 °C and − 20 °C. Non-soaked (NS) and Celest Top-coated seeds were used as controls. **C** Shoot length. **D** Total root lengths. Vertical bars represent the standard deviation. Red and black asterisks indicate statistically significant increase and decrease, respectively, in shoot and root length as compared to the control NS (RT) (*p* < 0.05; unpaired t test, GraphPad)

Germination analysis of imbibed seeds showed variability associated with the storage condition and treatment. Accordingly, as shown in Fig. 2A, for seeds imbibed in water or WB at various concentrations, storage at room temperature (RT) or 4 °C achieved the highest germination percentages as compared to storage at − 20 °C. Germination percentages in these treatments were indistinguishable from that of control seeds, namely NS and Celest Top stored at RT. Wheat seeds imbibed in GP extracts showed essentially similar germination percentages at all storage conditions.

### Effects on number of seminal roots

The number of seminal roots was strongly influenced by both the seed treatment and the storage condition (Fig. 2B). A higher proportion of 4 and 5 seminal roots is demonstrated in seedlings derived from seeds treated with Celest Top and WB100 stored for 1 month at RT or at 4 °C. Closer analysis revealed that the proportion of 1–3 SRs was 50 to 81% in seedlings derived from WB50, WB25, GP100, GP50, GP25, and NS treatments irrespective of storage conditions (Fig. 2B). The highest proportion of seedlings showing 4–5 SRs was found under Celest Top and WB100 treatments under RT and 4 °C storage conditions; under 4 °C, 84% of seedlings derived from WB100 stored at 4 °C displayed the highest 4–5 SRs. This might suggest that WB contains growth factors (e.g., auxin) that promote SR initiation and growth. Figure 2C illustrates that nearly all treatments exhibited longer shoots in comparison to the control NS (RT), regardless of the storage conditions. Conversely, the storage conditions had a significant effect on root length. In this regard, seeds stored at -20 °C resulted in statistically significant shorter roots for WB at all concentrations when compared to the control NS (Fig. 2D).

### Effects of 6-months storage on seed germination and post-germination growth

**Fig. 3:**
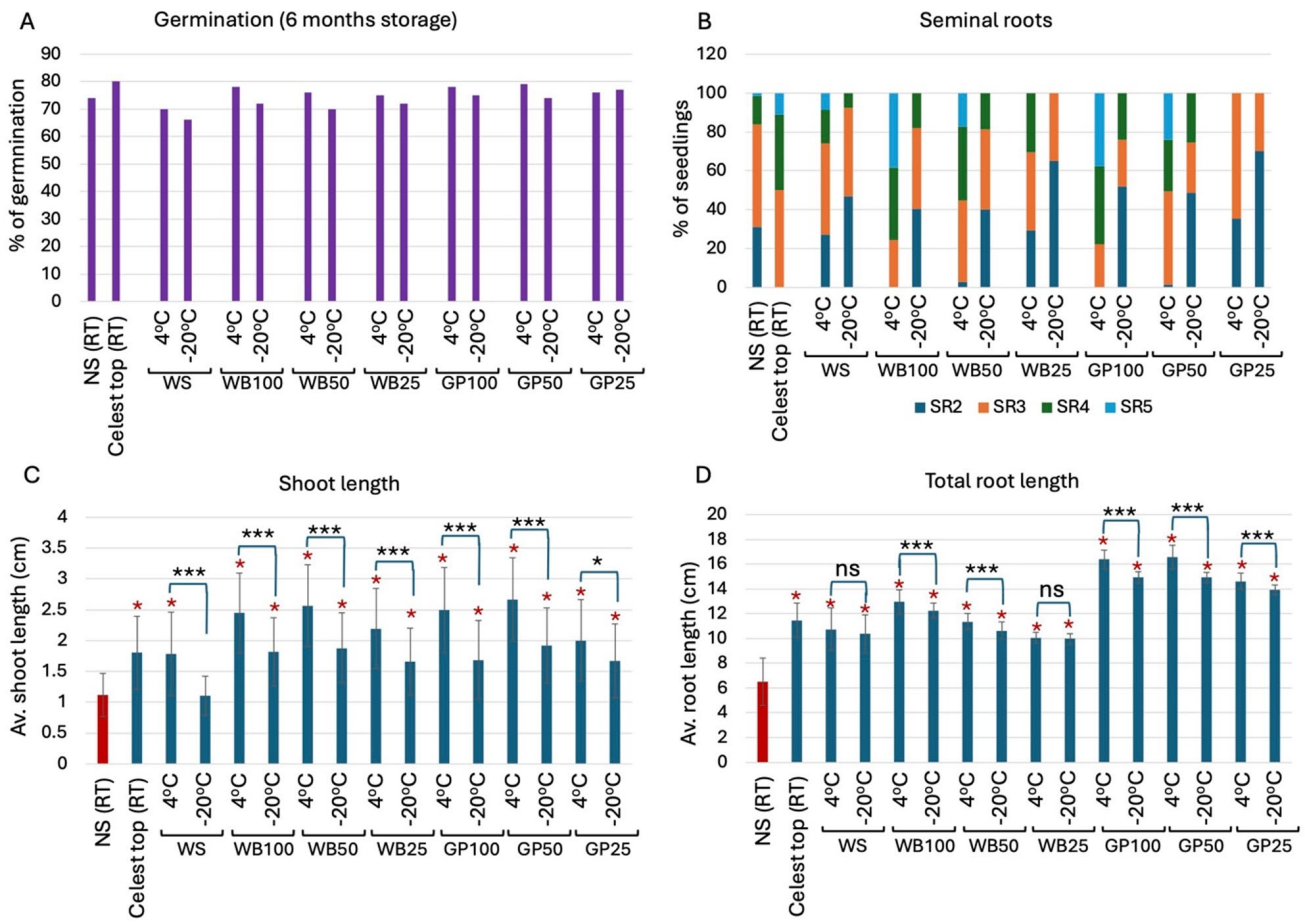
Effects of AIBW (WB and GP) on seedling performance after 6 months storage at 4 °C and − 20 °C. Non-soaked (NS) and Celest Top-coated seeds were used as controls. **A** Germination percentages recorded after 72 h. **B** Effect of AIBWs on (SR) production. The proportion of wheat seedlings developing various numbers of seminal roots (SR2 to SR5) is presented in a stacked column. **C** Shoot length. **D** Total root lengths. Vertical bars represent the standard deviation. Red asterisks indicate statistically significant increase in shoot and root length as compared to the control NS (RT) (*p* < 0.05; unpaired t test, GraphPad). Black asterisks denote statistical significance between storage temp. of 4 °C and − 20 °C in each treatment. ***p < 0.0001; *p < 0.01

Notably, seeds stored at room temperature were excluded due to insect infestation, so only seeds stored at 4 °C and − 20 °C were evaluated. Germination percentages after 72 h are presented in Fig. 3A. Thus, germination analysis of imbibed seeds stored for 6 months at 4 °C or − 20 °C showed essentially similar germination percentages when compared to one month of storage, ranging from 66 to 80% germination. A slight reduction in germination was observed in seeds stored at a temperature of -20 °C for all treatments.

The number of SRs was strongly influenced by both the seed treatment and the storage condition (Fig. 3B). The least number of SRs was generated by seeds treated with WB25 and preserved at 4 °C and by GP25 preserved at 4 °C or − 20 °C, yielding a maximum of 3 SRs. Apparently, seedlings obtained from NS seeds preserved at RT, WB25 at 4 °C, and seeds preserved at -20 °C across all treatments yielded a moderate number of SRs, reaching a maximum of 4 SRs. The highest proportion of seedlings generating 4 and 5 SRs was obtained by Celest Top-coated seeds and by AIBW-treated seeds kept at 4 °C. Accordingly, the proportion of seedlings with 4 and 5 SRs was 50%, 55%, and 50% in Celest Top, WB50, and GP50; the highest proportion of seedlings with 4 and 5 SRs was found in WB100 (~ 75%) and GP100 (~ 78%). This indicates that WB and GP at high concentrations contain growth factors (e.g., auxin) that promote SR initiation and growth. Indeed, the analysis of WB and GP revealed a relatively high level of the auxin indole acetic acid (IAA, Priyanka et al. 2024), which is known to induce root formation (reviewed in Overvoorde et al. 2010).

**Fig. 4:**
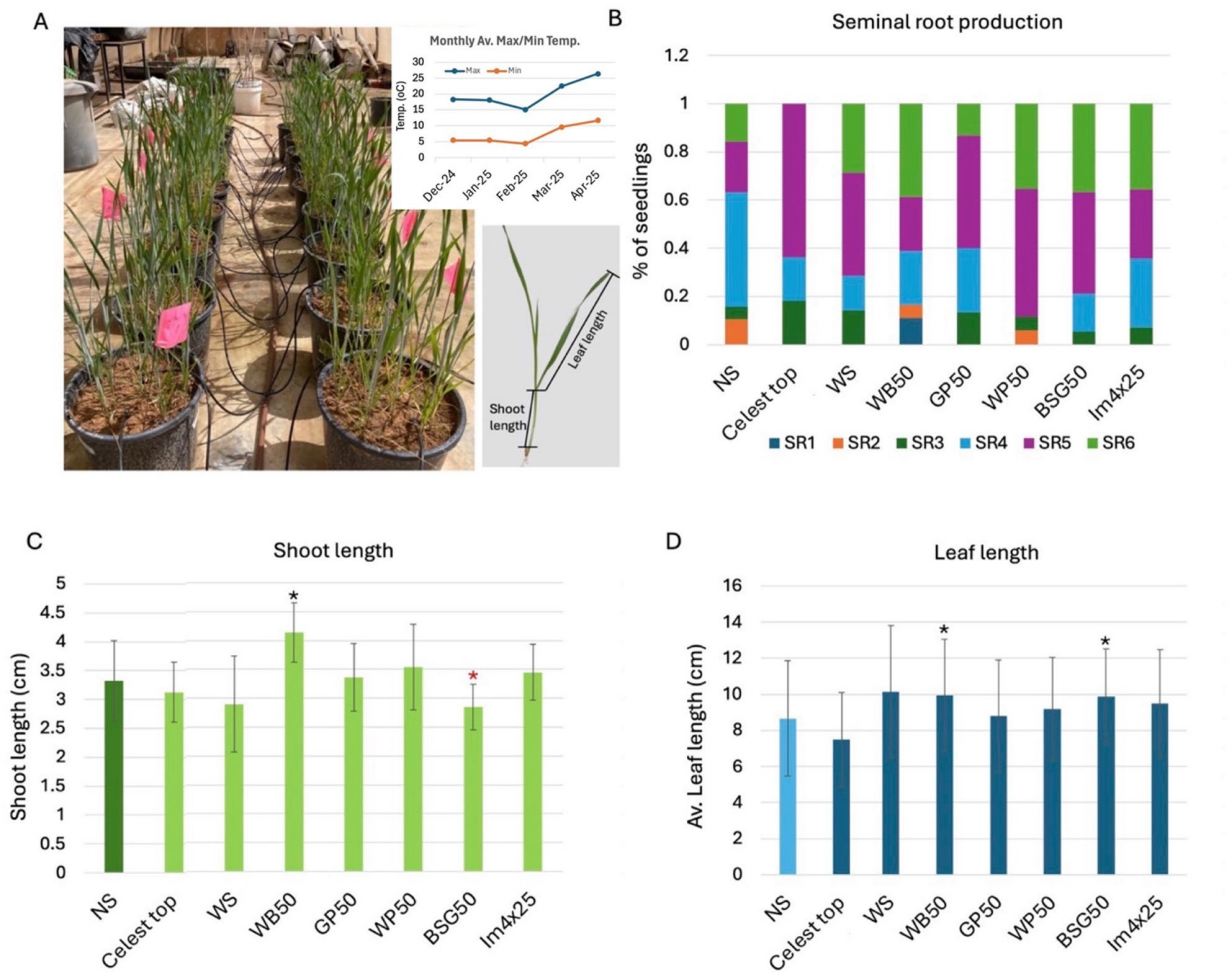
Mid-experiment analysis evaluating the effect of seed treatments on growth and development of wheat plants. **A** The net house view of the wheat plants at the time of sampling. The max/min temperature during the growing season is given on the upper right panel (Data for Sede Boqer station obtained from the Israel Meteorological Service (https://ims.gov.il/he/data_gov#). Bottom right panel depicts the measured segments of the plant. **B** Effect of AIBWs on (SR) production. The proportion of wheat seedlings developing various numbers of seminal roots (SR1 to SR6) is presented in a stacked column. **C** Shoot length. **D** Average leaf length. Vertical bar represents the standard deviation. Black and red asterisks indicate statistically significant increase and decrease, respectively, as compared to the NS (*p* < 0.05, unpaired t test, GraphPad)

The analysis of shoot and root lengths showed that essentially all treatments exhibited statistically significant longer shoots and roots in comparison to the control NS (RT) (red asterisks in Fig. 3C). However, for all treatments, storage at 4 °C resulted in statistically significantly longer shoots as compared to storage at − 20 °C (Fig. 4C, black asterisks). WB and GP at all concentrations, which were stored at 4 °C, displayed a statistically significant increase in shoot length as compared to Celest Top and WS. All treatments showed longer roots than NS, while GP-treated seeds at all concentrations and irrespective of storage condition had longer roots than all other treatments (Fig. 3D). Yet, all treatments except WS and WB25 showed significantly longer roots in seedlings derived from seeds stored at 4 °C as compared to − 20 °C (Fig. 3D).

### The impact of seed treatment in AIBW extracts on the growth of wheat plants: nethouse experiments

**Fig. 5:**
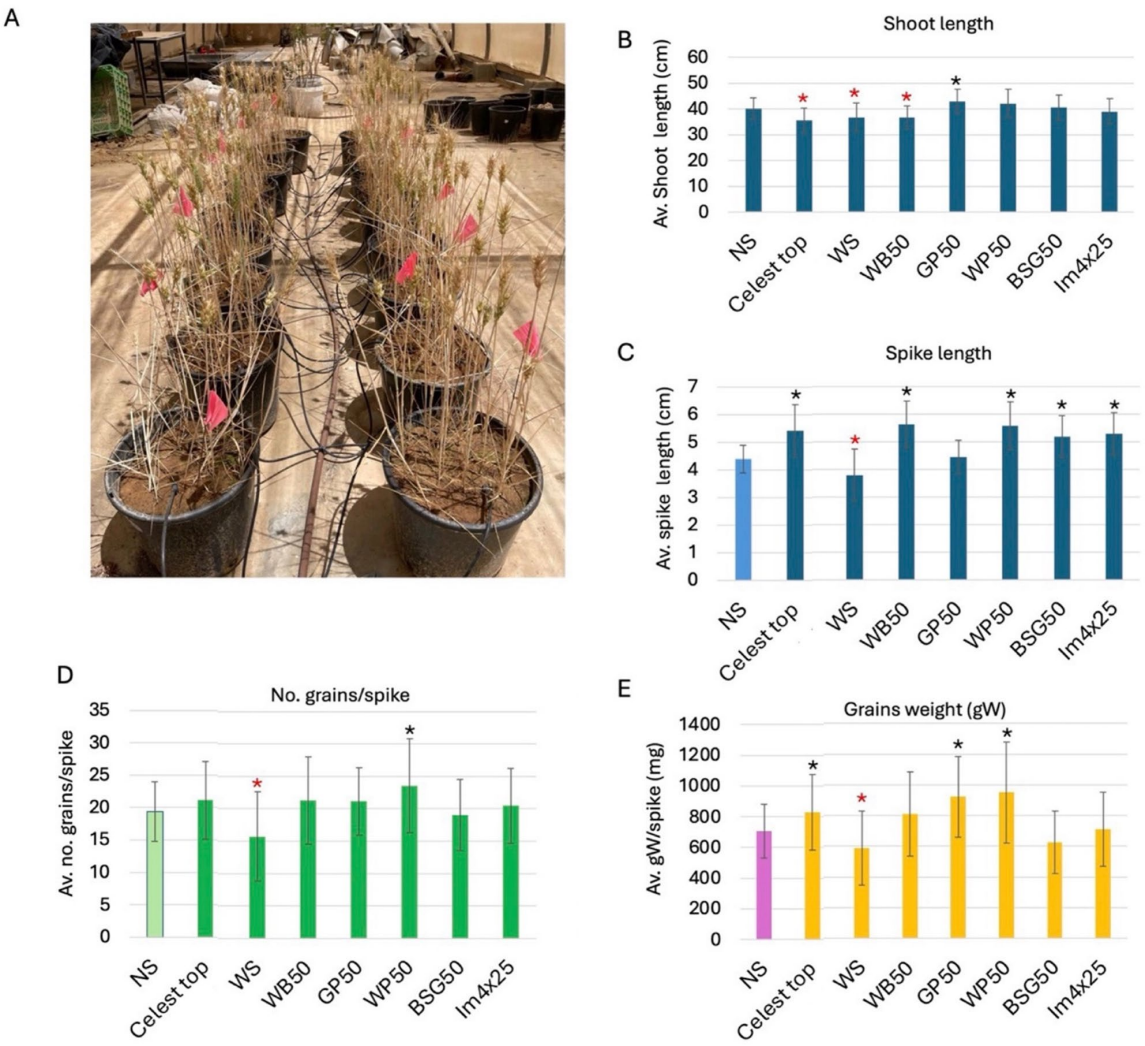
Analysis of net house experiment at harvest, evaluating the effect of seed treatments on growth and development of wheat plants. **A** The net house view of the wheat plants at the time of harvest. **B** Average shoot length. Measurement was taken from soil surface to the base of the spike. **C** Average spike length. **D** Average number of seeds per spike. **E** Average seed weight (gW) per spike. Vertical bar represents the standard deviation. Black and red asterisks indicate statistically significant increase and decrease, respectively, as compared to the NS (*p* < 0.05, unpaired t test, GraphPad)

This experiment evaluated the impact of nine seed treatments, including AIBW extracts and mixed AIBW (Im4 × 25), as compared to reference seeds, NS, WS, and Celest Top. Data collection occurred at mid-experiment (Fig. 4A) *via* destructive sampling for measuring early growth parameters and at final harvest for yield components (Fig. 5A).

Mid-experiment analysis of plant growth parameters such as shoot length, leaf length, and the number of seminal roots revealed distinct responses to the applied seed treatments in comparison with non-soaked plants (NS) serving as the baseline reference. An analysis of the number of seminal roots produced by wheat plants (Fig. 4B) indicated that AIBW-treated seeds yielded plants with the highest proportion of 5 and 6 SRs as compared to the control NS. Most prominent were WP50-imbibed seeds, in which 88% of seedlings produced 5 and 6 SRs. Also, seeds treated with BSG50, WS, Im4 × 25, WB50, and GP50 resulted in a high proportion (> 60%) of plants carrying 5 and 6 SRs; no seedlings with 6 SRs were found under Celest Top treatment. The control NS seeds exhibited the lowest percentage of plants with 5 and 6 SRs (37%).

Shoot length (Fig. 4C) was slightly affected by seed treatments, with WB50-treated seeds displaying statistically significant longer shoots (4.1 cm) and BSG50 shorter shoots (2.8 cm) as compared to control NS seeds (3.3 cm). The average length of the leaf (Fig. 4D) was determined by sampling 3 leaves from the top of each plant. Thus, the average leaf length ranges from 7.5 cm to 10 cm, where WB50 and BSG50 display statistically significantly longer leaves as compared to NS.

The final analysis of the net house experiment (Fig. 5A) details the effects of treatments on key growth and yield parameters at harvest, including shoot length, spike length, number of seeds per spike, and total seed weight, as well as metabolic analysis of the seeds. The Control NS served as the comparative reference.

At harvest, shoot length exhibited varied responses (Fig. 5B). Plants derived from GP50-treated seeds significantly increased shoot length, averaging 43 cm, a substantial improvement over the NS at 40 cm (*p* = 0.0090). Conversely, Celest Top (35.7 cm), WB50 (36.6 cm), and the WS (36.8 cm) all resulted in statistically significant reductions in shoot length (*P* = 0.0002, *P* = 0.0019, and *P* = 0.0084, respectively). Other treatments showed no significant differences.

Several treatments significantly influenced spike length (Fig. 5C). Statistically significant increases in spike length (*p* < 0.05) in comparison to NS were found in plants derived from treated seeds, including Celest Top, WB50, WP50, BSG50, and Im4 × 25. In contrast, the WS significantly reduced spike length as compared to NS (*p* = 0.0042).

The number of seeds per spike showed certain variation, though it was not well correlated with the spike length. Accordingly, besides WS and WP with reduced and increased seed numbers, respectively, as compared with NS, all other treatments had no significant effect on seed number per spike (Fig. 5D). Finally, the weight of seeds per spike was measured (Fig. 5E), showing a significant increase in weight of Celest Top, GP50, and WP50 and a reduction in WS as compared to NS.

### Seeds derived from AIBW-treated plants display variation in metabolic profile

**Fig. 6:**
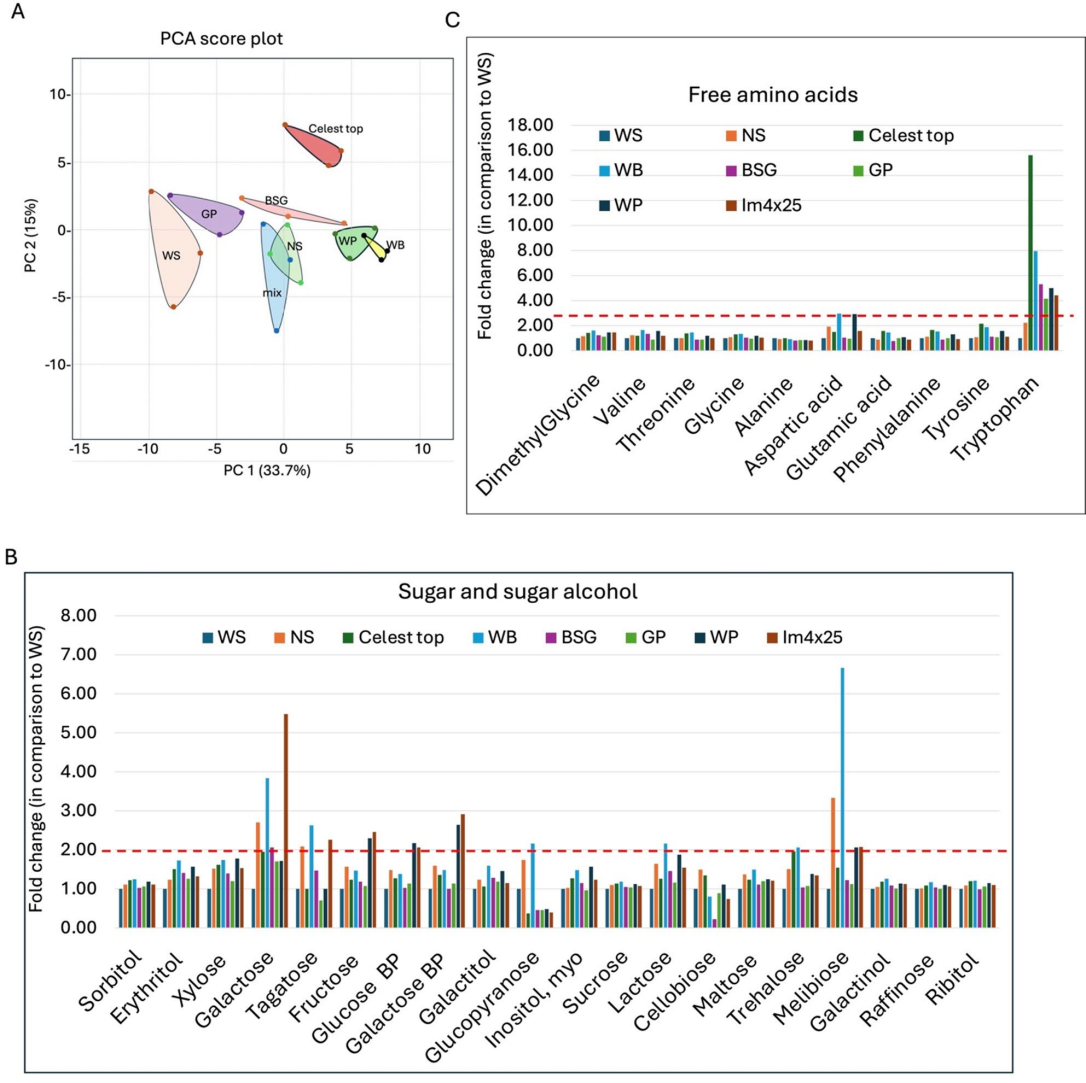
Variation in metabolite profiles in seeds produced from plants emerged from AIBW-treated wheat seeds. **A** PCA score plot showing clustering of metabolites according to treatments. Mix refers to Im4 × 25. Fold change in metabolite levels in comparison to WS-treated seeds. **B** Sugar and sugar alcohols. **C** Free amino acids. Red lines represent the 2-fold increase in metabolite level as compared to WS. Note sorbitol and ribitol were used as internal standards

We compared the metabolic profiles of seeds derived from plants whose seeds were treated with AIBWs. Analysis of primary metabolites was performed using GC-MS (in 4 repeats) and identified 79 primary metabolites (supplementary Table S1). A principal component analysis (PCA) separated metabolites according to treatments, showing that treating seeds with AIBW or Celest Top has a long-lasting effect on the metabolites accumulated in the newly produced seeds (Fig. 6A). Careful analysis of metabolite groups revealed that among sugars and sugar alcohols (supplementary Fig. S1), sucrose is the most abundant in wheat seeds, followed by raffinose, galactinol, and maltose (supplementary Fig. S1A). Yet, their accumulation in wheat seeds was not affected by seed treatment (Fig. 6B). Other sugars and sugar alcohols affected by seed treatments (i.e., WP and Im4 × 25 mix) include galactose BP, fructose, and glucose BP that displayed over a 2-fold increase as compared to control WS (Fig. 6B). Other affected sugars include galactose, tagatose, glucopyranose, trehalose, lactose, and melibiose, showing over a 2-fold increase as compared to control WS, particularly under seeds treated with WB (Fig. 6B). The most prominent amino acid affected by all seed treatments is tryptophan (supplementary Fig. S1B), an essential amino acid whose level was increased between 4- and 15-fold as compared to control WS; Celest Top-coated seeds showed the highest effect (Fig. 6C).

Other differentially expressed metabolites include lumichrome, tartaric acid, and urea, whose relative content was increased in treated seeds as compared to WS (Supplementary Fig. S2A, C). Accordingly, lumichrome was accumulated over 2-fold in NS, WB, WP, and Im4 × 25; tartaric acid was increased over 30-fold in WP and Im4 × 25; and urea was increased over 10-fold in NS, WB, WP, and Im4 × 25 (Supplementary Figs. S2B, S2D).

### The impacts of seed coating with GP substances on germination and post-germination growth

**Fig. 7:**
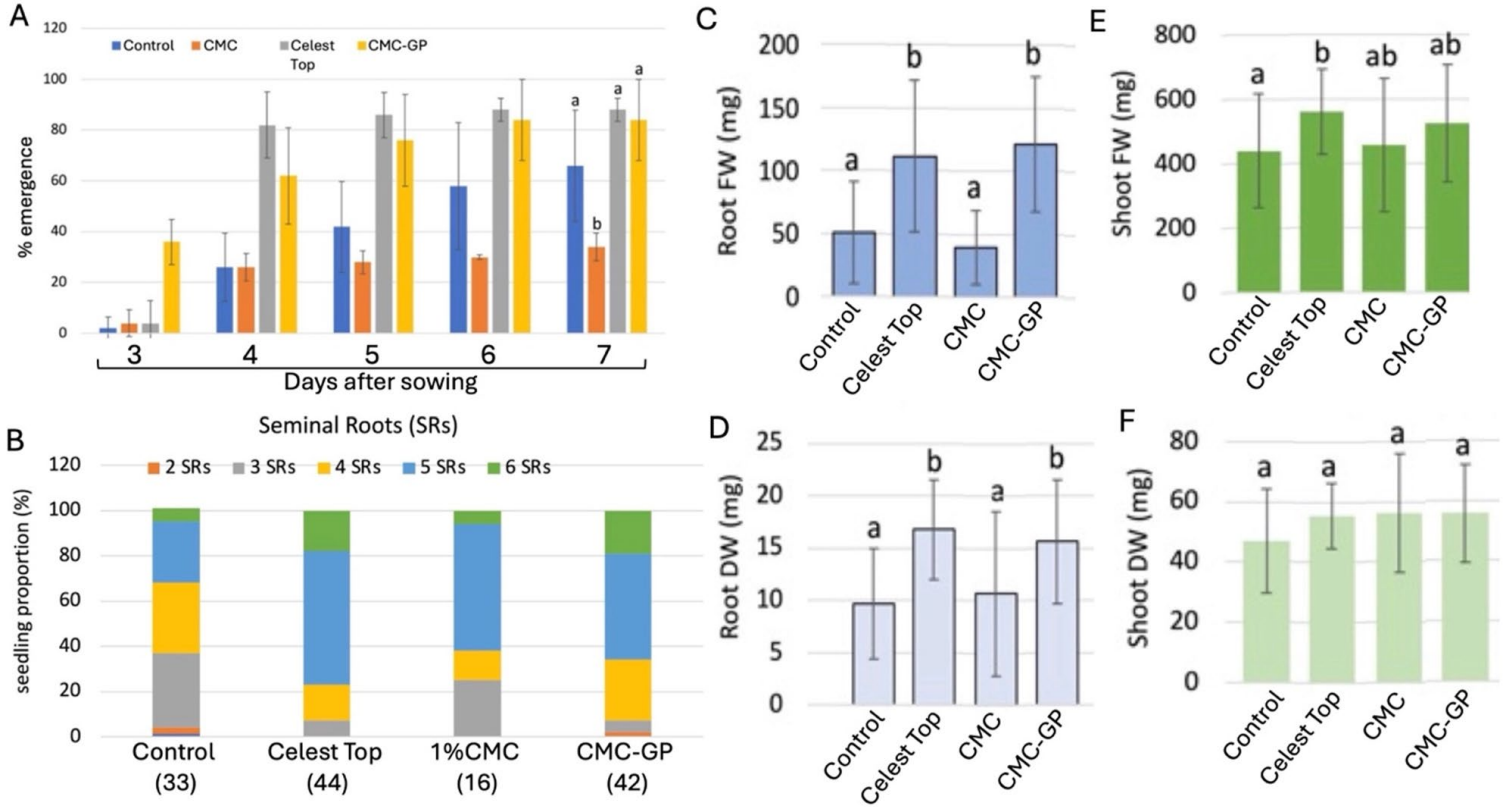
Effect of coating with GP substances (CMC-GP) on wheat seedling emergence and performance. **A** Emergence of seedlings was inspected daily and recorded from day 3 to day 7 after sowing. Statistical analysis was performed between coated seeds and control uncoated seeds. Each treatment was performed in 5 replicates, each containing 10 seeds. Vertical bars represent the standard deviation. Different letters (7th day) indicate statistically significant differences between control and each type of coated seeds (*p* < 0.01, One-Way ANOVA Calculator, Including Tukey HSD, Social Science Statistics). **B** The proportion of seedlings developing various numbers of seminal roots (SRs). **C**–**F** Effects of seed treatments on post germination growth of wheat seedlings. Measurements were taken at 14 days after sowing. **C** Root fresh weight (FW), **D** Root dry weight (DW), **E** Shoot FW, and **F** Shoot DW. Each treatment was performed in 5 replicates, each containing 10 seeds. Vertical bars represent the standard deviation. Different letters indicate statistically significant differences between treatments (*p* < 0.01, One-Way ANOVA Calculator, Including Tukey HSD, Social Science Statistics)

We examined coating seeds with substances derived from garlic peel (GP) extract (after lyophilization) on germination and post-germination growth. We used nature-sourced polysaccharide carboxymethylcellulose (CMC), a water-soluble polymer used as a thickening agent, stabilizer, and emulsifier in foods and for improving the storability of fresh-cut fruits (Shebis et al. 2022). Accordingly, wheat seeds were coated with 1% (w/v) CMC (CMC) or with 1% CMC containing 10 mg/ml GP substances (CMC-GP). Coated seeds were analyzed for their germination/emergence and post-germination growth and compared to uncoated seeds (control) or to the Celest Top-coated seeds. Accordingly, we have run experiments in 0.5 L pots to examine the effect on germination/emergence of wheat seedlings and on post-germination growth. Results presented in Fig. 7A show the rate of seedling emergence in a course of 7 days after sowing. Emergence was apparent already after 3 days, where seeds coated with CMC-GP were emerging first, demonstrating 36% of seedling emergence. On the 7th day after sowing, the percentage of seedling emergence for CMC was very poor at 34%, compared to 66% for the control uncoated seeds, 88% for Celest Top, and 84% for CMC-GP. On the 7th day, the differences between the control, Celest Top, and GP were not statistically significant.

Post-germination growth of wheat seedlings was examined 14 days after sowing. We first monitored the number of seminal roots (SRs) produced on wheat seedlings under various treatments (Fig. 7B). Apparently, seedlings derived from Celest Top and CMC-GP-coated seeds displayed a high proportion of seedlings with more than 4 SRs (77 and 67%, respectively) as compared to uncoated seeds (33% of seedlings), suggesting that CMC-GP and Celest Top may contain root-inducing factors (e.g., auxin). Although CMC treatment showed a high proportion (62%) of seedlings with 5 and 6 SRs, the low emergence of seedlings (16 out of 50) under this treatment makes comparison inappropriate.

We also measured the root and shoot fresh and dry weights of seedlings after 14 days. Results showed (Fig. 7C–F) a significant effect of Celest Top and CMC-GP on root performance, bringing about an increase in root fresh (Fig. 7C) and dry (Fig. 7D) weights as compared to uncoated and CMC-treated seeds. We could not observe a major effect on shoot performance (Fig. 7E, F), and shoot fresh and dry weights were essentially indistinguishable between treatments.

## Discussion

Seeds are fundamental to agriculture, and their vitality at the time of sowing influences crop establishment, resilience, and ultimately the yield. For many years, seed treatments, especially chemical coatings, have been employed to enhance seed performance by protecting against pathogens and promoting germination. Nevertheless, coating with hazardous chemicals (e.g., pesticides, fungicides, etc.) may exert toxic effects on humans and animals (Margaoan et al. 2025; Schmuck and Lewis 2016), highlighting the urgent need for safer and sustainable alternatives (Durgadevi et al. 2025). Presently, the agri-food sector generates millions of tons of organic byproducts and wastes annually, much of which remains underutilized or discarded inappropriately, despite being abundant in bioactive compounds such as antimicrobial compounds, antioxidants, phenolics, organic acids, and phytohormones (Sadh et al. 2018; Kaza et al. 2018; Naqvi et al. 2020; Jackowski et al. 2020). Valorization of AIBWs as inputs in agriculture represents a powerful strategy within the circular bioeconomy, reducing waste burdens while enhancing crop productivity (Apprich et al. 2014; Prückler et al. 2014; Meena et al. 2025).

In the present study we evaluated four abundant, safe, and eco-friendly AIBWs, namely, WB, GP, BSG, and WP, as ‘green’ treatments for wheat seeds, with the specific goal of determining whether these residues could substitute for the commercial hazardous chemical coating (e.g., Celest Top). Using multiple assays, pot experiments, and net-house trials, seeds imbibed in AIBWs or coated with CMC-GP consistently displayed improved post-germination performance compared with untreated control NS seeds. These improvements were evident in several key traits, including shoot and root lengths, seminal root production, and overall biomass accumulation, all of which are critical indicators of seed vigor and early crop establishment. Importantly, the results presented here demonstrated that WB- and GP-treated seeds frequently matched or even surpassed Celest Top-coated seeds in their effects, particularly in stimulating seminal root production as demonstrated in Petri dish assays. One of the most striking outcomes of this study was the strong stimulation of seminal root number and elongation in wheat seeds treated with WB and GP. Seminal roots are the primary determinants of early nutrient and water uptake in wheat, which directly affects plant vigor, reproduction, and final yield (Smith and De Smet 2012). While untreated control (NS) seeds typically produced fewer seminal roots, WB- and GP-treated seeds consistently exhibited enhanced root proliferation, either equal to or greater than the performance of Celest Top. This indicates that WB and GP at high concentrations contain growth factors (e.g., auxin) that promote SR initiation and growth. Indeed, the analysis of WB and GP revealed a relatively high level of the auxin, indole acetic acid (IAA, Priyanka et al. 2024), which is known to induce root formation (reviewed in Overvoorde et al. 2010).

The longevity of treated seeds is essential for practical applications. We showed that storage at room temperature is possible for short-term storage (up to 1 month), but long-term storage (6 months) at room temperature may not be practical due to insect infestation and other potential seed damages that occur during storage (Zhang et al. 2021; Ranganathan and Groot 2023). Indeed, orthodox seeds (e.g., wheat seeds) displayed prolonged viability when stored in cool and dry conditions, while at higher temperatures the storability of the seeds is significantly diminished (Ellis and Hong 2006). Storage experiments revealed that seeds treated with WB and GP retained higher vigor and higher seminal root production as compared to NS. Thus, their performance at RT and 4 °C was not only significantly better than the untreated control (NS) but also comparable to Celest Top, confirming that natural seed treatments can preserve seed quality as effectively as synthetic coatings. The inferior performance of seeds stored at − 20 °C can be attributed to freezing-induced cellular injury often derived from the production of ice crystals inside and outside the cells that could rupture the cell membrane and/or due to the induction of oxidative damage induced by reactive oxygen species (ROS) (Arora 2018; Bailly 2004; Ranganathan and Groot 2023). Deep-freeze storage may also destabilize bioactive metabolites delivered by AIBWs, reducing their protective function. We cannot exclude the possibility that the adverse effect of storage at -20 °C resulted from insufficient seed drying after imbibition.

The net house experiment highlighted the beneficial effect of wine pomace (WP) on various growth parameters of wheat, displaying the highest effect on SR production. Consequently, WP also showed beneficial effects on spike length, number of seeds per spike, and the average seed weight per spike. These effects clearly illustrated a significant connection between the impact of WP on seminal root production and its influence on reproduction; WP and GP produced yields comparable to Celest Top. The effect of AIBW on reproduction is also exhibited in the metabolite profile. Thus, the analysis of primary metabolites revealed variation in primary metabolite composition and levels among treated seeds. Particularly the increase in content of the amino acid tryptophan under all AIBW treatment, with Celest Top and WB showing the highest increase as compared to WS. Tryptophan is an essential amino acid that serves as a precursor to produce multiple bioactive molecules, including the neurotransmitter serotonin, the sleep-regulating hormone melatonin, and niacin (vitamin B3). Therefore, the application of AIBWs for seed treatment not only positively influences yield but also enhances the nutritional quality of the seeds, which can contribute to improved human health (Slominski et al. 2002; Friedman 2018; Kałużna-Czaplińska et al. 2019; Xue et al. 2023). Another beneficial metabolite whose level was increased particularly under WB and Im4 × 25 is galactose, an essential sugar for humans that functions as a precursor for various important macromolecules like glycoconjugates and galactolipids. Defects in galactose metabolism can lead to serious health conditions, such as galactosemia (Conte et al. 2021). Also lumichrome and tartaric acid showed an increase in levels as compared to WS. Lumichrome is a light-activated compound derived from riboflavin (vitamin B2) that can function as a photosensitizer to eliminate bacteria and as a drug against lung cancer cells (Chantarawong et al. 2019; Gjestvang Grønlien et al. 2023). Tartaric acid is an organic acid present in grapes, which contributes to the quality of wine, and is also found in other fruits. It is frequently utilized as an acidulant in various foods, beverages, and candies (Li et al. 2023). The benefits of AIBW treatments extended beyond germination and seedling establishment into the reproductive phase. Compared with the untreated control, all AIBWs improved spike length, seed number, and seed weight. This persistence can be explained by the induction of a ‘memory’ during imbibition that allows seedlings to change the pattern of gene expression, probably *via* epigenetic means, that persists throughout the life cycle of the plant. Thus, treated seeds could accumulate micro- and macro-elements, protective sugars (raffinose, galactinol), energy-related organic acids (citric acid, malic acid), and hormone-related metabolites that remained influential during later stages of development (Zhang et al. 2022; Chen et al. 2023; Priyanka et al. 2025). Such metabolic and epigenetic reprogramming has been described as a hallmark of seed treatment responses, enabling plants to better withstand stresses and maintain productivity (Paparella et al. 2015; Jisha et al. 2013; Chaves and Oliveira 2004).

## Conclusions

This research illustrates that AIBWs serve as effective and sustainable treatments for seeds, enhancing seminal root growth, storage efficiency, and overall yield. AIBW substances can be utilized for efficient seed treatments either as water-soluble extracts through imbibition or by coating with biopolymers such as CMC. Compared with the controls, all treatments enhanced vigor, while WB, GP, and WP consistently matched or even exceeded the chemical coating Celest Top. WP contributed profoundly to reproduction, which is associated with increased production of SRs. Overall, AIBWs offer low-cost, eco-friendly alternatives to chemical seed coatings while contributing to waste valorization within the circular economy. With their abundance and consistent effectiveness, especially WB and WP, they represent a practical innovation for sustainable agriculture and can be used as effective substitutes for the hazardous chemical seed coating.

## Author contributions

Jayaseelan Joy Jacklin: Writing—original draft, Writing—review and editing, Investigation, Methodology, Visualization. Emilly Draru: Writing—review and editing, Investigation, Methodology, Formal analysis, Visualization. Govindegowda Priyanka: Writing—review and editing, Investigation, Methodology, Formal analysis, Visualization. Keerthana Yeduguru Reddy: Writing—review and editing, Methodology. Nurit Novoplansky: Writing—review and editing, Project administration, Methodology, Formal analysis. Ilan Chertok: Writing—review and editing, Investigation, Methodology. Elena Poverenov: Writing—review and editing, Funding acquisition, Conceptualization. Gideon Grafi: Writing—original draft, Writing—review and editing, Funding acquisition, Conceptualization, Visualization, Supervision, Formal analysis.

## Supplementary Information

Below is the link to the electronic supplementary material.

Supplementary Material 1

Supplementary Material 2

## Data Availability

Data will be made available on request.
