# Supplementary material for: Eco-friendly alternatives to seed chemical coating: agro-industrial byproducts as seed treatments with long-term effects on growth and reproduction
Source: Bioresour Bioprocess. 2026 Jan 24;13(1):4. doi: 10.1186/s40643-025-01000-5 (PMC12830535; doi:10.1186/s40643-025-01000-5)
Supplement: Supplementary file 2 — Supplementary Material 2 [file 40643_2025_1000_MOESM2_ESM.docx]

Supplementary figures

**Eco-friendly alternatives to seed chemical coating: agro-industrial byproducts as seed treatments with long-term effects on growth and reproduction**

Joy Jacklin Jayaseelan^1^, Emilly Draru^1^, Govindegowda Priyanka^1^, Keerthana Yeduguru Reddy^1^, Nurit Novoplansky^1^, Ilan Chertok^2,3^, Elena Poverenov^2^, Gideon Grafi^1^*


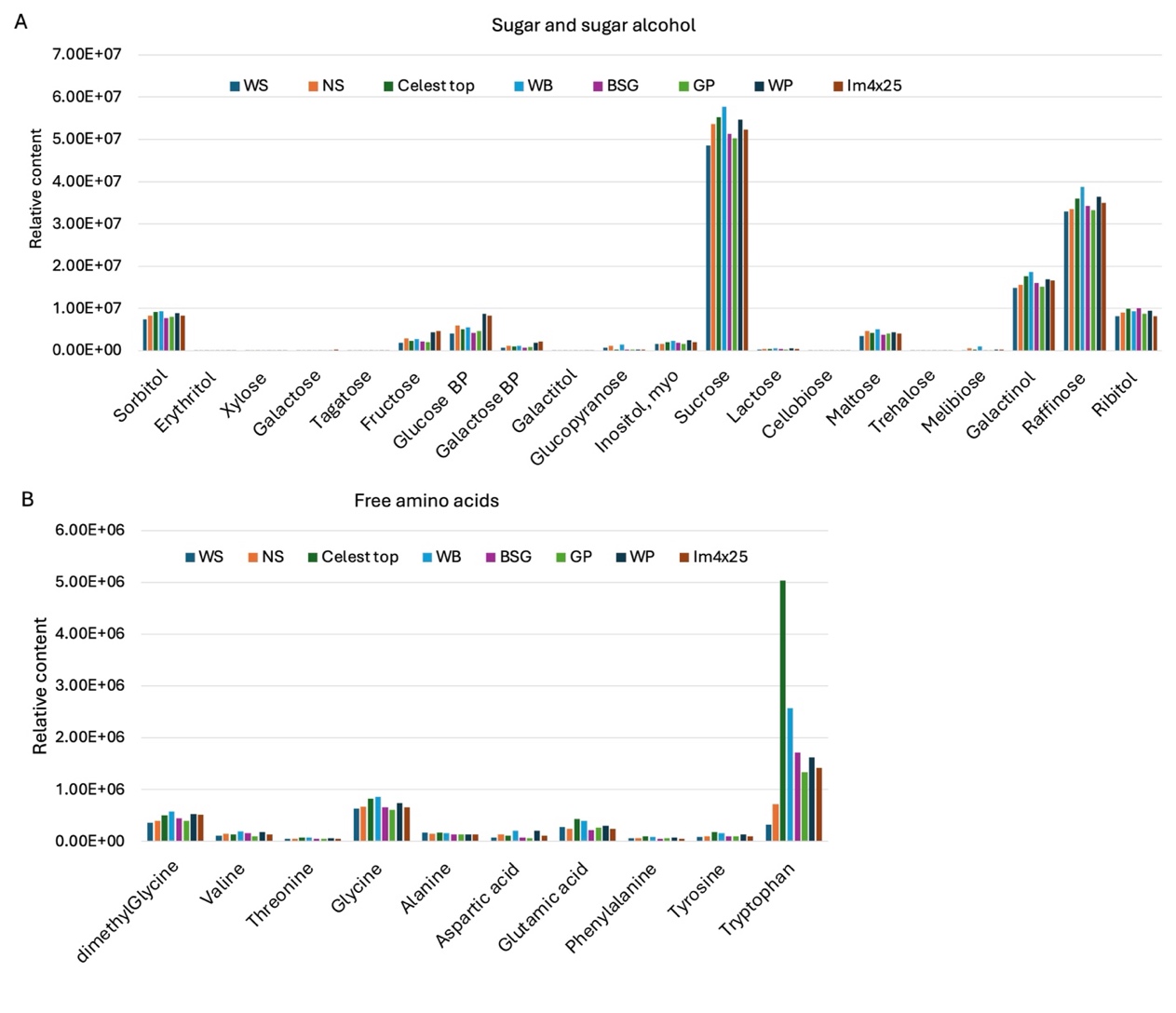


Fig. S1. Seed treatments affect metabolic profiles of the newly produced wheat seeds. Seeds derived from WS, Celest top and AIBW-treated seeds were subjected to primary metabolite analysis. (A) The realtive content of sugars and sugar alcohols in the newly produced seeds. (B) The relative content of free amino acids. Note, the high accumulation of tryptophan under all seed treatments. Sorbitol and ribitol are the internal standards.


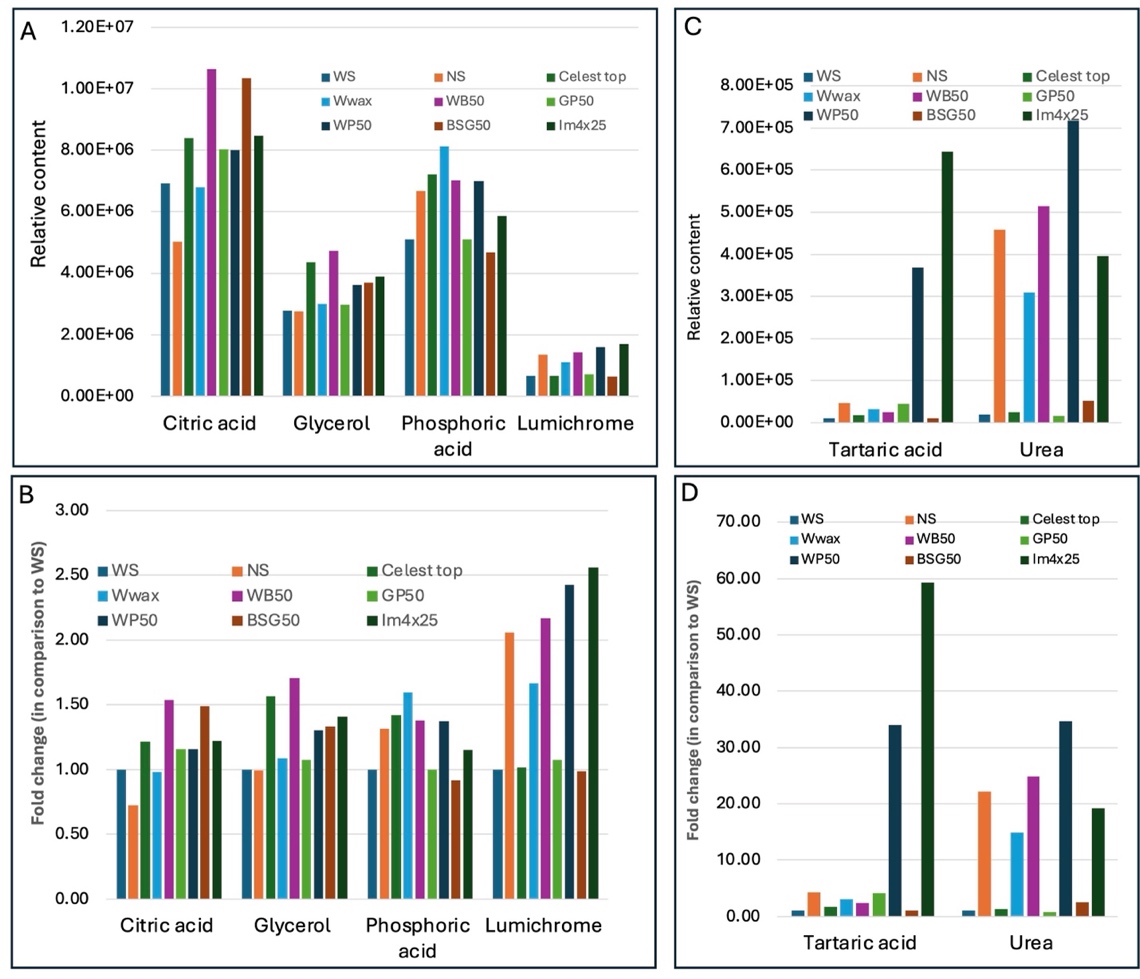


Fig. S2. Effect of seed treatments on various metabolites identified in the newly produced wheat seeds. (A, C) Relative content of various metabolites in seeds derived from AIBW and control plants. (B, D) Fold change of metabolite level in comparison to WS.
